# Supplementary material for: Complex‐centric proteome profiling by SEC‐SWATH‐MS
Source: Mol Syst Biol. 2019 Jan 14;15(1):e8438. doi: 10.15252/msb.20188438 (PMC6346213; doi:10.15252/msb.20188438)
Supplement: Supplementary file 8 — Dataset EV7 [file MSB-15-e8438-s008.zip › feature_plots_string/Q9Y296.pdf]

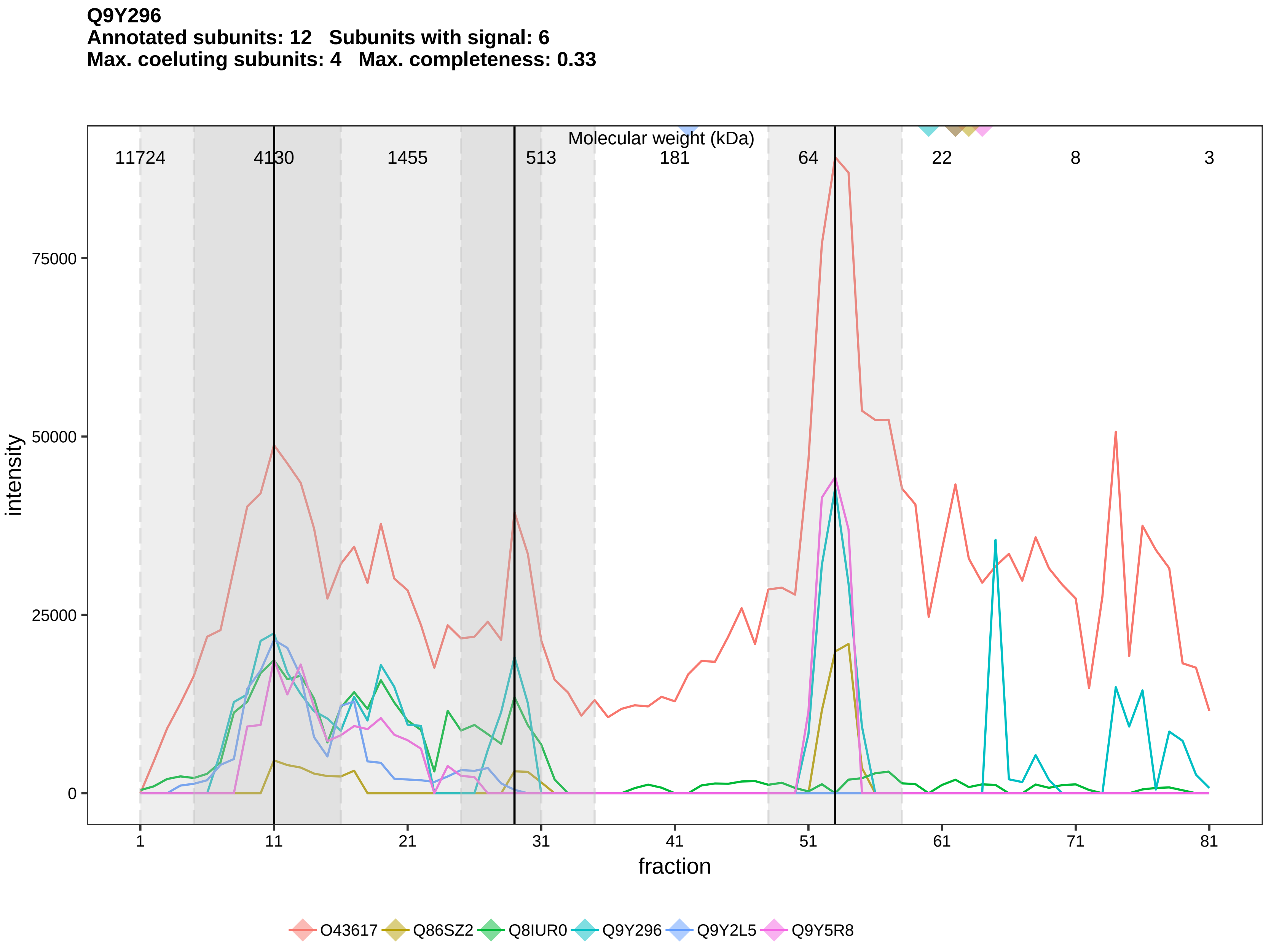

Q9Y296
Annotated subunits: 12   Subunits with signal: 6
Max. coeluting subunits: 4   Max. completeness: 0.33
Molecular weight (kDa)
11724
4130
1455
513
181
64
22
8
3
75000
50000
25000
0
intensity
1
11
21
31
41
51
61
71
81
fraction
O43617
Q86SZ2
Q8IUR0
Q9Y296
Q9Y2L5
Q9Y5R8
